# Genome-wide association study of metabolic syndrome in Korean populations

**DOI:** 10.1371/journal.pone.0227357

**Published:** 2020-01-07

**Authors:** Seung-Won Oh, Jong-Eun Lee, Eunsoon Shin, Hyuktae Kwon, Eun Kyung Choe, Su-Yeon Choi, Hwanseok Rhee, Seung Ho Choi

**Affiliations:** 1 Department of Family Medicine, Healthcare System Gangnam Center, Seoul National University Hospital, Seoul, South Korea; 2 DNA Link, Inc., Seoul, South Korea; 3 Department of Family Medicine, Seoul National University Hospital, Seoul, South Korea; 4 Department of Surgery, Healthcare System Gangnam Center, Seoul National University Hospital, Seoul, South Korea; 5 Department of Internal Medicine, Healthcare System Gangnam Center, Seoul National University Hospital, Seoul, South Korea; Graduate School of Medicine, University of the Ryukyus, JAPAN

## Abstract

Metabolic syndrome (MetS) which is caused by obesity and insulin resistance, is well known for its predictive capability for the risk of type 2 diabetes mellitus and cardiovascular disease. The development of MetS is associated with multiple genetic factors, environmental factors and lifestyle. We performed a genome-wide association study to identify single-nucleotide polymorphism (SNP) related to MetS in large Korean population based samples of 1,362 subjects with MetS and 6,061 controls using the Axiom^®^ Korean Biobank Array 1.0. We replicated the data in another sample including 502 subjects with MetS and 1,751 controls. After adjusting for age and sex, rs662799 located in the *APOA5* gene were significantly associated with MetS. 15 SNPs in *GCKR*, *C2orf16*, *APOA5*, *ZPR1*, and *BUD13* were associated with high triglyceride (TG). 14 SNPs in *APOA5*, *ALDH1A2*, *LIPC*, *HERPUD1*, and *CETP*, and 2 SNPs in *MTNR1B* were associated with low high density lipoprotein cholesterol (HDL-C) and high fasting blood glucose respectively. Among these SNPs, 6 TG SNPs: rs1260326, rs1260333, rs1919127, rs964184, rs2075295 and rs1558861 and 11 HDL-C SNPs: rs4775041, rs10468017, rs1800588, rs72786786, rs173539, rs247616, rs247617, rs3764261, rs4783961, rs708272, and rs7499892 were first discovered in Koreans. Additional research is needed to confirm these 17 novel SNPs in Korean population.

## Introduction

Metabolic syndrome (MetS) is defined as a cluster of metabolic abnormalities, including abdominal obesity, dyslipidemia, high blood glucose levels, and high blood pressure. Using the definition of the joint scientific statement on MetS [1], its prevalence was 34.2% in US adults and has been reported to increase [2]. In a Korean nationwide survey, 29% of male and 32.9% of female had MetS [3]. MetS is associated with an increased risk of type 2 diabetes, cardiovascular disease, cancer and death [4]. Moreover, individual components of MetS are important risk factors for cardiovascular diseases and are targets for therapeutic intervention. Multiple genetic loci associated with MetS and its components have been identified by genome-wide association studies (GWAS) [5]. Almost all these loci were reported initially in European ancestry populations, and many studies on Asians have been published recently. However, few such genetic studies have been performed, especially on Koreans. Herein, we conducted a GWAS on a Korean population. The aim of this study was to identify single-nucleotide polymorphism (SNP) that could be associated with MetS components in a Korean population.

## Materials and methods

### Study subjects

The subjects of our study included Korean adults who had undergone a routine health check-up program in the Seoul National University Hospital Healthcare System Gangnam Center from January 2014 to December 2014. They completed a self-administered questionnaire, which included questions on previous medical history and health related behavior. Patients were excluded from the study when they did not agree to participate in this study, had thyroid functional diseases, took medication or treatment for weight control, or had comorbidity such as myocardial infarction, cerebrovascular diseases, and cancers. After obtaining informed consent, 9,676 individuals donated blood samples, and their blood samples were stored at a biorepository. Anthropometric measurements and laboratory tests were conducted as part of a general health check-up, and genomic DNA was extracted from peripheral blood leukocytes of all participants using the QuickGene DNA whole-blood kit with QuickGene-610 L equipment (Fujifilm, Tokyo, Japan) according to standard protocols. The Institutional Review Board of the Seoul National University Hospital approved the storage of biospecimens with informed consent (IRB number 1103-127-357). We used the biospecimens retrospectively. The board approved this study protocol (IRB number 1504-004-659), and the informed consent was waived by the board.

### Biochemical measurements

Eligible participants completed a questionnaire that included demographic factors, comorbidities, and medication for conditions, including hypertension, diabetes mellitus, and hypercholesterolemia. Blood glucose, triglyceride (TG), and high density lipoprotein cholesterol (HDL-C) levels were measured after 12 hours of fasting by using an automated analyzer (Architect c8000; Toshiba Inc., Tokyo, Japan). Height and body weight were assessed after wearing light hospital gowns, and body mass index (BMI) was calculated based on the ratio of body weight to the square of height (kg/m^2^). Waist circumference (WC) was obtained in the midpoint of the iliac ridge and the lower end of the rib using a measuring tape. An automated sphygmomanometer was used for blood pressure measurement after enough resting time. We used the following criteria for the definition of MetS proposed by the International Diabetes Federation’s criteria for the South Asian ethnic group [6]. MetS was diagnosed if more than 3 of the following indications were present: (1) systolic blood pressure (SBP) ≥ 130 mm Hg or diastolic blood pressure (DBP) ≥ 85 mm Hg or currently on hypertension medication, (2) TG ≥ 150 mg/dL, (3) HDL-C level < 40 mg/dL for men and < 50 mg/dL for women, (4) fasting blood glucose (FBG) level ≥ 100 mg/dL or currently on diabetes medication, and (5) WC ≥ 90 cm for men and ≥ 80 cm for women.

### Genotyping and quality control

Genomic DNA was separated from venous blood samples, and 200 ng of genomic DNA was genotyped using Affymetrix Axiom^™^ KORV1.0–96 Array (Affymetrix, Santa Clara, CA, USA). The PLINK program (version 1.9; Free Software Foundation Inc., Boston, MA, USA) was used for quality control procedures. Samples matching any of the following criteria were excluded: (1) sex inconsistencies or (2) call rate up to 97%. SNPs were filtered if (1) the call rate was less than 95%, (2) the minor allele frequency was up to 1%, or (3) there was a significant deviation from the Hardy-Weinberg equilibrium permutation test (P < 1×10^−4^). After quality control, 584,061 autosomal SNPs remained for the association analysis. Genotype data were produced using the Korean Chip (K-CHIP) available through the K-CHIP consortium. K-CHIP was designed by Center for Genome Science, Korea National Institute of Health, Korea (4845–301, 3000–3031).

### Statistical analysis

A total of 584,061 SNPs that passed the quality control was used for GWAS. Baseline characteristics of the study population were presented as mean with standard deviation for continuous variables and number with proportion for categorical variables. We compared the clinical characteristics between subjects with and without MetS using a t-test for continuous variables and a chi-square test for categorical variables. We carried out a case-control study between the individuals with MetS components and without MetS components. Multivariate linear regressions, adjusted for the effects of age and body mass index via an additive model, were used to further investigate the influence of SNPs on Mets components. PLINK software was used for the statistical analysis and to draw the Manhattan plot of −log_10_. The results were verified using discovery and replication sets. We divided the enrolled population into two groups on the basis of the time of enrollment. Samples enrolled from January 2014 to October 2014 were considered the discovery set (n = 7,423), and those enrolled in the subsequent periods were used as the replication set (n = 2,253). SNPs that had a P-value of less than 10^−8^ in the discovery set were re-evaluated for replication. P-values less than 0.05 were considered significant in the replication set.

We calculated an inflation factor which was estimated from the mean of the χ2 tests generated on all SNPs that were tested. The inflation factors in the discovery set were close to 1 for MetS and its components (S1 Table). Principal component analysis (PCA) for our Korean population and 1000 genome phase 3 data revealed the expected ancestry with individuals of east Asian origin (KOR: Korean, CHB: Chinese, JPT: Japanese) which were clustered, those of European origin (CEU) and those of African origin (YRI) (S Fig). Genotype imputation was performed with the software IMPUTE2. 1000 genome phase 3 data was used as a reference panel. Only SNPs imputed with high confidence (Info Score ≥0.8) were chosen for this study. After that, we compared the SNPs which was reported to be related to MetS components in a recent Korean study using Korean Association REsource (KARE) and Health EXAminee (HEXA) cohort data [7] with our results.

## Results

The mean age of the study subjects was 50.6 years in the discovery set and 51.0 years in the replication set. Table 1 shows the clinical characteristics of the study subjects including the MetS components: BMI, WC, SBP, DBP, fasting glucose, TG, and HDL-C. Among 7,423 in the discovery set, 1,362 (18.3%) were included as MetS cases and among the 2,253 replication set, 502 (22.3%) were included as MetS cases.

**Table 1:** Baseline characteristics of the participants.

	Discovery set (N = 7423)			Replication set (N = 2253)		
	MetS (N = 1362)	No MetS (N = 6061)	P-value	MetS (N = 502)	No MetS (N = 1751)	P-value
Age (years)	53.5 ± 9.5	49.9 ± 10.2	<0.01	52.5 ± 9.5	50.6 ± 9.9	<0.01
Sex (male)	1100 (80.8%)	3216 (53.1%)	<0.01	408 (81.3%)	953 (54.4%)	<0.01
BMI (kg/m^2^)	26.1 ± 2.8	22.4 ± 2.7	<0.01	26.5 ± 2.7	22.7 ± 2.7	<0.01
WC (cm)	91.7 ± 6.7	80.5 ± 7.8	<0.01	92.9 ± 7.1	81.6 ± 7.9	<0.01
SBP (mmHg)	123.9 ± 12.3	113.5 ± 12.7	<0.01	124.6 ± 12.7	113.8 ± 13.0	<0.01
DBP (mmHg)	83.2 ± 9.1	74.3 ± 9.8	<0.01	83.3 ± 9.6	74.9 ± 9.7	<0.01
Fasting glucose	112.0 ± 21.6	95.2 ± 13.5	<0.01	116.0 ± 31.1	96.5 ± 13.6	<0.01
Triglyceride	180.7 ± 96.5	91.3 ± 53.2	<0.01	181.1 ± 117.6	90.5 ± 48.2	<0.01
HDL-cholesterol	46.0 ± 9.7	55.6 ± 11.9	<0.01	45.2 ± 9.0	54.9 ± 11.6	<0.01
Current smoking	323 (23.7%)	858 (14.2%)	<0.01	107 (21.3%)	281 (16.0%)	<0.01
Alcohol (drink/week)	15.9 ± 17.1	9.6 ± 13.7	<0.01	17.2 ± 19.6	9.8 ± 13.7	<0.01
Physical activity (METS)	960.7 ± 2011.0	936.9 ± 1690.6	0.65	733.1 ± 1867.4	858.8 ± 2266.1	0.26
Diabetes medication	128 (9.4%)	119 (2.0%)	<0.01	50 (10.0%)	41 (2.3%)	<0.01
Hypertension medication	492 (36.1%)	544 (9.0%)	<0.01	168 (33.5%)	145 (8.3%)	<0.01

Values are presented as mean ± standard deviation or number (%). P values are calculated from t-test for continuous variables or from chi-square test for categorical variables. MetS, metabolic syndrome; BMI, body mass index; WC, waist circumference; SBP, systolic blood pressure; DBP, diastolic blood pressure; HDL, high density lipoprotein

After adjusting for age and sex, rs662799 located in the *APOA5* gene were significantly associated with MetS in our population (P = 2.85 × 10^−10^). Association between rs662799 and MetS was maintained in the replication set (P = 3.19 × 10^−3^) (Table 2). We identified 15 SNPs with a significant influence on hypertriglyceridemia (≥ 150 mg/dL). Rs780092, rs780093, rs780094, rs1260326, and rs1260333 in the *GCKR* gene; rs1919127 and rs1919128 in the *C2orF16* gene; rs662799, rs2075291, and rs2266788 in the *APOA5* gene; rs603446 and rs964184 in the *ZPR1* gene; and rs11216126, rs1558861, and rs2075295 in the *BUD13* gene were associated with hypertriglyceridemia. These 15 SNPs were also validated in the replication set (Table 3). 14 SNPs were associated with low HDL-C (< 40 mg/dL for men and < 50 mg/dL for women). Rs662799 and rs2075291 in the *APOA5* gene; rs4775041, rs10468017, and rs1800588 in the *ALDH1A2* or *LIPC* gene; and rs72786786, rs173539, rs247616, rs247617, rs3764261, rs4783961, rs708272, rs7499892, and rs2303790 in the *HERPUD1* or *CETP* gene were identified and validated (Table 4). Two SNPs, rs10830962 and rs10830963 in the *MTNR1B* gene, were associated with high FBG (≥ 100 mg/dL or currently on diabetes medication) (Table 5). Manhattan plot and quantile-quantile plot of the GWAS were drawn using data from discovery and replication set (S2 and S3 Figs). Additionally, rs6589566 in the *ZPR1* gene, which had been previously identified in KARE and HEXA cohort data [7], was also associated with hypertriglyceridemia in the genotype imputation (S2 Table).

**Table 2:** Significant variants associated with metabolic syndrome.

Chr	SNP	Position	Gene	M	Discovery set			Replication set		
					MAF	OR	P	MAF	OR	P
					(case / control)	(95% CI)		(case / control)	(95% CI)	
11	rs662799	116663707	APOA5	G	0.345 / 0.288	1.346	2.85×10^−10^	0.334 / 0.290	1.268	3.19×10^−3^
						(1.227–1.476)			(1.083–1.485)	

Chr, chromosome; rs number, SNP ID in dbSNP database; M, minor allele; MAF, minor allele frequency; OR, odds ratio; CI, confidence interval, respectively

**Table 3:** Significant variants associated with hypertriglyceridemia (TG ≥150 mg/dL).

Chr	SNP	Position	Gene	M	Discovery set			Replication set		
					MAF	OR	P	MAF	OR	P
					(case / control)	(95% CI)		(case / control)	(95% CI)	
2	rs780092	27743154	GCKR	G	0.275 / 0.334	0.752	4.82×10^−9^	0.279 / 0.336	0.753	1.19×10^−3^
						(0.683–0.827)			(0.635–0.894)	
2	rs780093	27742603	GCKR	C	0.395 / 0.471	0.730	2.55×10^−12^	0.398 / 0.474	0.721	5.47×10^−5^
						(0.669–0.797)			(0.615–0.845)	
2	rs780094	27741237	GCKR	C	0.397 / 0.472	0.736	6.49×10^−12^	0.400 / 0.475	0.723	5.91×10^−5^
						(0.674–0.803)			(0.617–0.847)	
2	rs1260326	27730940	GCKR	C	0.385 / 0.460	0.731	3.89×10^−12^	0.392 / 0.463	0.729	1.07×10^−4^
						(0.669–0.799)			(0.622–0.856)	
2	rs1260333	27748624	GCKR	G	0.392 / 0.468	0.734	5.20×10^−12^	0.392 / 0.472	0.704	1.75×10^−5^
						(0.672–0.802)			(0.600–0.827)	
2	rs1919127	27801493	C2orf16	T	0.417 / 0.477	0.776	1.18×10^−8^	0.420 / 0.491	0.755	3.67×10^−4^
						(0.711–0.846)			(0.646–0.881)	
2	rs1919128	27801759	C2orf16	A	0.415 / 0.476	0.772	7.39×10^−9^	0.419 / 0.491	0.754	3.65×10^−4^
						(0.707–0.843)			(0.646–0.881)	
11	rs662799	116663707	APOA5	G	0.391 / 0.278	1.770	4.97×10^−34^	0.389 / 0.277	1.732	8.49×10^−11^
						(1.614–1.94)			(1.467–2.045)	
11	rs2075291	116661392	APOA5	A	0.117 / 0.068	1.940	3.67×10^−19^	0.106 / 0.069	1.739	6.93×10^−5^
						(1.678–2.243)			(1.324–2.284)	
11	rs2266788	116660686	APOA5	G	0.273 / 0.209	1.482	9.26×10^−15^	0.281 / 0.207	1.538	4.03×10^−6^
						(1.342–1.637)			(1.281–1.846)	
11	rs603446	116654435	ZPR1	T	0.191 / 0.242	0.726	6.24×10^−9^	0.178 / 0.231	0.705	5.48×10^−4^
						(0.651–0.809)			(0.578–0.860)	
11	rs964184	116648917	ZPR1	G	0.271 / 0.208	1.479	1.47×10^−14^	0.279 / 0.205	1.552	2.93×10^−6^
						(1.339–1.634)			(1.291–1.866)	
11	rs2075295	116628401	BUD13	C	0.404 / 0.466	0.755	4.56×10^−10^	0.424 / 0.480	0.793	4.04×10^−3^
						(0.691–0.825)			(0.677–0.929)	
11	rs11216126	116617240	BUD13	C	0.150 / 0.205	0.666	1.34×10^−11^	0.177 / 0.206	0.808	3.70×10^−2^
						(0.592–0.749)			(0.661–0.987)	
11	rs1558861	116607437	BUD13	C	0.275 / 0.213	1.463	5.85×10^−14^	0.278 / 0.212	1.463	4.37×10^−5^
						(1.325–1.616)			(1.219–1.756)	

Chr, chromosome; rs number, SNP ID in dbSNP database; M, minor allele; MAF, minor allele frequency; OR, odds ratio; CI, confidence interval, respectively

**Table 4:** Significant variants associated with low HDL-C (HDL-C level <40 mg/dL for men and<50 mg/ dL for women).

Chr	SNP	Position	Gene	M	Discovery set			Replication set		
					MAF	OR	P	MAF	OR	P
					(case / control)	(95% CI)		(case / control)	(95% CI)	
11	rs662799	116663707	APOA5	G	0.371 / 0.284	1.472	2.26×10^−16^	0.365 / 0.283	1.444	9.37×10^−6^
						(1.342–1.614)			(1.227–1.699)	
11	rs2075291	116661392	APOA5	A	0.121 / 0.068	1.915	9.28×10^−9^	0.124 / 0.065	2.042	3.89×10^−8^
						(1.659–2.209)			(1.583–2.633)	
15	rs4775041	58674695	ALDH1A2	C	0.167 / 0.217	0.717	2.28×10^−8^	0.157 / 0.229	0.634	8.72×10^−6^
						(0.639–0.806)			(0.519–0.775)	
15	rs10468017	58678512	ALDH1A2	T	0.167 / 0.215	0.726	7.27×10^−8^	0.154 / 0.225	0.624	6.83×10^−6^
						(0.646–0.816)			(0.508–0.767)	
15	rs1800588	58723675	ALDH1A2	T	0.371 / 0.429	0.778	5.62×10^−8^	0.375 / 0.414	0.842	3.45×10^−2^
			LIPC			(0.710–0.852)			(0.718–0.988)	
16	rs72786786	56985514	HERPUD1	A	0.132 / 0.185	0.663	1.65×10^−10^	0.128 / 0.182	0.645	1.27×10^−4^
			CETP			(0.584–0.752)			(0.516–0.807)	
16	rs173539	56988044	HERPUD1	T	0.205 / 0.258	0.732	1.13×10^−8^	0.219 / 0.262	0.796	1.38×10^−2^
			CETP			(0.658–0.815)			(0.665–0.955)	
16	rs247616	56989590	HERPUD1	T	0.108 / 0.176	0.562	1.29×10^−16^	0.113 / 0.177	0.587	8.36×10^−6^
			CETP			(0.490–0.644)			(0.464–0.742)	
16	rs247617	56990716	HERPUD1	A	0.106 / 0.174	0.557	7.70×10^−17^	0.113 / 0.175	0.594	1.38×10^−5^
			CETP			(0.486–0.639)			(0.470–0.752)	
16	rs3764261	56993324	HERPUD1	A	0.105 / 0.173	0.553	5.27×10^−17^	0.112 / 0.173	0.598	1.97×10^−5^
			CETP			(0.481–0.635)			(0.472–0.757)	
16	rs4783961	56994894	HERPUD1	A	0.201 / 0.256	0.732	9.93×10^−9^	0.213 / 0.249	0.817	3.37×10^−2^
			CETP			(0.657–0.814)			(0.678–0.985)	
16	rs708272	56996288	CETP	A	0.337 / 0.395	0.777	6.09×10^−8^	0.350 / 0.392	0.839	3.21×10^−2^
						(0.709–0.851)			(0.715–0.985)	
16	rs7499892	57006590	CETP	T	0.210 / 0.162	1.371	1.57×10^−8^	0.209 / 0.157	1.441	2.06×10^−4^
						(1.229–1.53)			(1.188–1.748)	
16	rs2303790	57017292	CETP	G	0.018 / 0.048	0.349	5.31×10^−11^	0.023 / 0.056	0.409	1.81×10^−4^
						(0.255–0.478)			(0.256–0.653)	

Chr, chromosome; rs number, SNP ID in dbSNP database; M, minor allele; MAF, minor allele frequency; OR, odds ratio; CI, confidence interval, respectively

**Table 5:** Significant variants associated with high fasting blood glucose (FBG level ≥100 mg/ dL or currently on diabetes medication).

Chr	SNP	Position	Gene	M	Discovery set			Replication set		
					MAF	OR	P	MAF	OR	P
					(case / control)	(95% CI)		(case / control)	(95% CI)	
11	rs10830962	92698427	MTNR1B	G	0.473 / 0.423	1.277	1.15×10^−10^	0.478 / 0.436	1.251	1.12×10^−3^
						(1.186–1.376)			(1.093–1.43)	
11	rs10830963	92708710	MTNR1B	G	0.469 / 0.409	1.329	8.03×10^−14^	0.47 / 0.425	1.26	6.30×10^−4^
						(1.233–1.432)			(1.104–1.438)	

Chr, chromosome; rs number, SNP ID in dbSNP database; M, minor allele; MAF, minor allele frequency; OR, odds ratio; CI, confidence interval, respectively

## Discussion

We identified SNPs and genomic regions associated with MetS and its components in a Korean population. In this study, 15 SNPs were reported to be associated with TG level. The SNP rs662799 in the *APOA5* gene was associated with increased risk of MetS and its components, especially elevated TG and low levels of HDL-C. Two other SNPs in *APOA5*, rs2266788 and rs2075291, were also associated with elevated TG level. *APOA5* gene on chromosome 11q23.3 is known to be associated with dyslipidemia, which is a component of MetS, and a risk of coronary heart disease [5, 8]. A number of SNPs of *APOA5* associated with TG and HDL-C levels have been reported [9]. Among these SNPs, rs662799, which is located in the promotor region of the *APOA5* gene, was associated with TG levels and coronary heart disease in a Japanese and a Chinese population [10, 11]. Association between rs662799 and elevated TG level was validated in Koreans [12, 13]. The *APOA5* 3'-UTR variant, rs2266788, is associated with TG levels through downregulation of *APOA5* [14]. This SNP was associated with TG, HDL-C level, and MetS in a European [15] and a Chinese [16] population. In a previous Korean study including 1,193 men, rs2266788 showed marginal association with TG and MetS (P = 0.0027; OR, 1.402) [9]. Rs2075291, a missense SNP in *APOA5*, is known to be rare in populations of European ancestry. This SNP was associated with HDL-C and TG levels in Chinese and Korean population in previous studies [17, 18].

Other variants of the *ZPR1* and *BUD13* genes at chromosome 11q23.3 are also known to be associated with serum lipid level. *ZPR1*, also known as *ZNF259*, encodes a zinc-finger protein, ZPR1. This protein is essential for the normal nuclear function during cell proliferation. Additionally, the promoter site of *ZPR1* binds peroxisome proliferator-activated receptor gamma (PPARG) proteins 1 and 2, which play a key role in insulin sensitivity and obesity [19, 20]. *BUD13* is one of the subunits of the RES complex, previously identified in yeast as a splicing factor affecting nuclear pre-mRNA retention [21]. The SNPs rs964184, rs603446, rs2075295, rs11216126, and rs1558861 in *ZPR1* and *BUD13* gene were associated with elevated TG level in this study. Although the association of the *ZPR1* and *BUD13* SNPs and serum lipid levels has been reported in the European and Asian population [22, 23], little is known about such an association in Korean populations. In a previous Korean study, rs603446 and rs11216126 were associated with elevated TG and HDL-C level, respectively [24]. However, no previous study has reported the association of rs964184, rs2075295, and rs1558861 with TG levels in Koreans.

In this study, the SNPs rs1260326, rs1260333, rs780092, rs780093, and rs780094 in the *GCKR* gene were associated with elevated TG level. *GCKR* at chromosome 2p23.3 encodes the glucokinase regulatory protein (GKRP), which modulates the activity of hepatic hexokinase and, thereby, gates the entry of glucose into the glycolytic and glycogen synthesis pathways. Variants of the gene encoding GKRP were found to have converse effects on TG and glucose metabolic traits [25]. Rs1260326, which is a non-synonymous variant in *GCKR*, and rs1260333, located downstream of *GCKR*, were reported to have inverse effects on TG and glucose levels in European descent populations [26–28]. The association between these two SNPs and TG level is validated in Chinese and Japanese [29, 30]. Moreover, TG-increasing alleles of *GCKR* variants rs1260326 and rs1260333 lowered insulin and HOMA-IR and reduced the risk of insulin resistance in Chinese [31]. The association between rs780092, rs780093, and rs780094 in *GCKR* and TG levels was also reported in previous European and Asian population studies [25, 32]. In a Korean study, as in our study, major allele carriers of rs780092 and rs780094 in *GCKR* had significantly higher serum TG levels compared to noncarriers [33]. To our knowledge, the present study is the first to report the effects of *GCKR* variants rs1260326 and rs1260333 on TG levels in a Korean population. In Korean populations, rs1260326 was associated with the risk of IFG and type 2 diabetes [34] and total and low-density lipoprotein (LDL) cholesterol in children [33]. Rs1919127 and rs1919128 at *C2orf16* gene on chromosome 2p23.3 were associated with elevated TG level in our study. Rs1919128 was associated with elevated TG level in a recent Korean study [18]. On the other hand, our study is the first to report that rs1919127 in *C2orf16* were associated with elevated TG level in Korean population.

Fourteen SNPs were reported to be associated with HDL-C level in this study. Three SNPs, rs4775041, rs10468017, and rs1800588 were located in *ALDH1A2* and *LIPC* gene on chromosome 15q21.3. *ALDH1A2* encodes the protein aldehyde dehydrogenase 1 family member A2. This enzyme catalyzes the synthesis of retinoic acid from retinaldehyde and retinoic acid, the active derivative of vitamin A, is a hormonal signaling molecule that functions in normal organ development [35]. Variants of the *ALDH1A2* gene was associated with lipid traits in previous several Asian studies [29, 36]. The *LIPC* gene provides instructions for making hepatic lipase, which helps with the conversion of very low-density lipoprotein (VLDL) to LDL. The enzyme also assists in transporting HDL that carries cholesterol and TG from the blood to the liver. Variants of the *LIPC* gene are well known to be associated with dyslipidemia [25, 29, 37]. However, our study is the first to report that these 3 SNPs are associated with HDL-C level in Korean. There are relatively few reports on the associations between these polymorphisms and plasma lipid concentrations in Asian individuals. Rs1800588 was associated with low HDL-C level in Chinese population [29, 38]. On the other hand, unlike our study, rs10468017 was not associated with dyslipidemia in another Chinese study, which matched 524 patients with hyperlipidemia with 621 normal subjects [39]. Nine SNPs, rs72786786, rs173539, rs247616, rs247617, rs3764261, rs4783961, rs708272, rs7499892, and rs2303790 are located in the cholesteryl ester transfer protein (*CETP*) or *HERPUD1* gene on chromosome 16q13. CETP is an enzyme responsible for moving cholesterol esters and TG between VLDL, LDL, and HDL. Low CETP levels promote HDL formation. Polymorphisms in the *CETP* gene, which result in reduced CETP expression, are associated with high plasma HDL-C level and a low prevalence of cardiovascular disease [40]. Of these SNPs, rs3764261 and rs2303790 were associated with HDL level in Japanese [41, 42] and rs708272 was associated with risk of coronary atherosclerosis in Chinese [43]. The association between rs2303790 and low HDL-C was confirmed in a recent Korean study [18]. Our study is the first to report the association between other eight SNPs and HDL-C in Korean.

Two SNPs, rs10830962 and rs10830963 in the *MTNR1B* gene were associated with high FBG in this study. The *MTNR1B* gene encodes one of the two known human melatonin receptors, the MT2, and it is highly expressed in beta cells. *MTNR1B* allele is known to be involved in the regulation of insulin secretion [44]. Previous Korean studies reported the association between rs10830962 and glucose level and gestational diabetes mellitus [45, 46]. Another study reported that rs10830963 was strongly associated with gestational diabetes mellitus in Korean women [47].

In this study, we could not find a SNP that was related to blood pressure in Korean. The mechanism of blood pressure control is not sufficiently explained by the genetic effect, and hypertension is caused by complex interactions between genetic and environmental factors. There have been several Korean studies that examined the interaction between SNPs and environmental factors on the risk of hypertension [48–50]. In this regard, further studies will be necessary.

Few previous studies have evaluated the association between genetic variants and MetS in Korean populations. In 2,657 MetS cases and 5,917 controls among the Korean Genome and Epidemiology study (KoGES) subjects, only two SNPs, rs11216126 and rs180349, were identified with significant p-values (< 5 × 10^−8^) [51]. Of these two SNPS, rs11216126 is also associated with elevated TG level in our study. Authors of the study emphasized that the multiple correction criteria of conventional GWASs for excluding false-positive loci could simultaneously discard many true-positive loci. In another Korean study, rs662799 of *APOA5* was significantly associated with regulated TG levels and MetS, like our study [13]. Participants of this study were only men; however, it was similar to our study in that the participants visited for a routine health check-up. The interaction between this SNP and health-related behaviors was also evaluated in this study, and the SNP showed interactions with alcohol drinking and physical activity. Thus, the results suggested that a strategy of prevention and treatment should be tailored to personal genotype and population. However, we could not evaluate the interaction between environmental and genetic factors. In the recent Korean study using KARE and HEXA cohort data, 21 including five new SNPs were replicated for MetS components [7]. Of these SNPs, rs11216126 and rs2303790 were also associated with TG level and HDL-C level in our study. Additionally, one SNP, rs6589566 in the *ZPR1* gene was additionally confirmed in the imputation analysis. Rs6589566 was also associated with TG levels, in Hispanic and European populations [52, 53].

In conclusion, we identified 15 TG SNPs, 14 HDL SNPs, and 2 FBG SNPs in this GWAS. Among these SNPs, 6 TG SNPs: rs1260326 and rs1260333 in *GCKR*, rs1919127 in *C2orf16*, rs964184 in *ZPR1*, and rs2075295 and rs1558861 in *BUD13* and 11 HDL SNPs: rs4775041, rs10468017, and rs1800588 in *ALDH1A2* and *LIPC* and rs72786786, rs173539, rs247616, rs247617, rs3764261, rs4783961, rs708272, and rs7499892 in *CETP* or *HERPUD1* were first discovered in Koreans. Additional research is needed to confirm these new SNPs.

## Supporting information

S1 Table Inflation factor for metabolic syndrome and its components in the discovery set. (DOCX)

S2 Table Association between SNPs identified in a recent Korean study and metabolic syndrome components in our study subjects. (DOCX)

S1 Fig Principal component analysis (PCA) for our Korean population and 1000 genome phase 3 data. (DOCX)

S2 Fig Manhattan plot for metabolic syndrome components. (DOCX)

S3 Fig Quantile-quantile plots of the association test results for metabolic syndrome components. (DOCX)

S1 Appendix The questionnaire for study subjects. (DOCX)
