# Supplementary material for: Genome-wide association study of metabolic syndrome in Korean populations
Source: PLoS One. 2020 Jan 7;15(1):e0227357. doi: 10.1371/journal.pone.0227357 (PMC6946588; doi:10.1371/journal.pone.0227357)
Supplement: S1 Table — (DOCX) [file pone.0227357.s001.docx]

**S1 Table. Inflation Factor for Metabolic Syndrome and Its Components in the Discovery Set**

| **Phenotype** | **inflation factor** |
| --- | --- |
| Metabolic syndrome | 1.0178 |
| Hypertriglyceridemia | 1.0202 |
| Low HDL-C | 1.0200 |
| High FBG | 1.0178 |
| High BP | 1.0158 |

HDL, high density lipoprotein; FBG, fasting blood glucose; BP, blood pressure, respectively
