# Supplementary material for: Genome-wide association study of metabolic syndrome in Korean populations
Source: PLoS One. 2020 Jan 7;15(1):e0227357. doi: 10.1371/journal.pone.0227357 (PMC6946588; doi:10.1371/journal.pone.0227357)
Supplement: S2 Table — (DOCX) [file pone.0227357.s002.docx]

**S2 Table. Association between SNPs identified in a Recent Korean Study [7] and Metabolic Syndrome Components in Our Study Subjects**

| SNP | Method | Hypertriglyceridemia | | | | Low HDL-C | | | | High FBG | | | | High BP | | | |
| --- | --- | --- | --- | --- | --- | --- | --- | --- | --- | --- | --- | --- | --- | --- | --- | --- | --- |
|  |  | Discovery | | Replication | | Discovery | | Replication | | Discovery | | Replication | | Discovery | | Replication | |
|  |  | OR  (95% CI) | P | OR  (95% CI) | P | OR  (95% CI) | P | OR  (95% CI) | P | OR  (95% CI) | P | OR  (95% CI) | P | OR  (95% CI) | P | OR  (95% CI) | P |
| rs10503669 | Chip | 0.712 (0.618-0.820) | 2.51🞨10^-6^ | 0.708 (0.546-0.919) | 9.51🞨10^-3^ | 0.721 (0.624-0.833) | 9.55🞨10^-6^ | 0.685 (0.531-0.884) | 3.66🞨10^-3^ | 1.008 (0.903-1.126) | 8.82🞨10^-1^ | 1.042 (0.854-1.27) | 6.86🞨10^-1^ | 0.957 (0.857-1.069) | 4.42🞨10^-1^ | 0.945 (0.772-1.159) | 5.91🞨10^-1^ |
| rs10849915 | Chip |  |  |  |  |  |  |  |  |  |  |  |  |  |  |  |  |
| rs11065756 | imputation | 0.873 (0.779-0.978) | 1.95🞨10^-2^ | 0.859 (0.703-1.05) | 1.37🞨10^-1^ | 1.146 (1.026-1.28) | 1.55🞨10^-2^ | 1.013 (0.836-1.227) | 8.95🞨10^-1^ | 0.867 (0.788-0.953) | 3.10🞨10^-3^ | 0.863 (0.732-1.018) | 7.98🞨10^-2^ | 0.842 (0.766-0.927) | 4.30🞨10^-4^ | 0.855 (0.723-1.01) | 6.56🞨10^-2^ |
| **rs11216126** | Chip | **0.666 (0.592-0.749)** | **1.34🞨10^-11^** | **0.808 (0.661-0.987)** | **3.70🞨10^-2^** | 0.823 (0.734-0.923) | 9.15🞨10^-4^ | 0.762 (0.624-0.932) | 8.18🞨10^-3^ | 0.985 (0.899-1.079) | 7.48🞨10^-1^ | 0.951 (0.81-1.116) | 5.39🞨10^-1^ | 1.032 (0.941-1.13) | 5.03🞨10^-1^ | 1.057 (0.899-1.241) | 5.03🞨10^-1^ |
| rs11986942 | imputation | 0.740 (0.661-0.828) | 1.71🞨10^-7^ | 0.810 (0.666-0.986) | 3.57🞨10^-2^ | 0.744 (0.663-0.835) | 4.69🞨10^-7^ | 0.821 (0.677-0.995) | 4.43🞨10^-2^ | 1.023 (0.935-1.119) | 6.21🞨10^-1^ | 1.034 (0.884-1.209) | 6.78🞨10^-1^ | 1.019 (0.931-1.114) | 6.83🞨10^-1^ | 1.073 (0.916-1.257) | 3.82🞨10^-1^ |
| rs12708980 | imputation | 0.988 (0.852-1.147) | 8.83🞨10^-1^ | 1.303 (1.01-1.68) | 4.15🞨10^-2^ | 1.399 (1.218-1.606) | 2.05🞨10^-6^ | 1.765 (1.39-2.24) | 3.14🞨10^-6^ | 0.990 (0.874-1.122) | 8.83🞨10^-1^ | 1.145 (0.916-1.429) | 2.34🞨10^-1^ | 1.052 (0.923-1.19) | 4.20🞨10^-1^ | 1.072 (0.857-1.341) | 5.40🞨10^-1^ |
| rs17410962 | imputation | 0.718 (0.624-0.826) | 4.09🞨10^-6^ | 0.713 (0.550-0.924) | 1.06🞨10^-2^ | 0.726 (0.629-0.838) | 1.33🞨10^-5^ | 0.681 (0.528-0.878) | 3.14🞨10^-3^ | 1.011 (0.906-1.128) | 8.46🞨10^-1^ | 1.057 (0.867-1.287) | 5.83🞨10^-1^ | 0.956 (0.856-1.068) | 4.28🞨10^-1^ | 0.942 (0.769-1.153) | 5.62🞨10^-1^ |
| rs17411031 | Chip | 0.738 (0.659-0.827) | 1.61🞨10^-7^ | 0.786 (0.645-0.958) | 1.74🞨10^-2^ | 0.734 (0.653-0.824) | 1.69🞨10^-7^ | 0.832 (0.687-1.008) | 6.04🞨10^-2^ | 1.027 (0.938-1.123) | 5.69🞨10^-1^ | 1.023 (0.874-1.196) | 7.78🞨10^-1^ | 1.021 (0.933-1.117) | 6.56🞨10^-1^ | 1.04 (0.887-1.219) | 6.27🞨10^-1^ |
| rs17411126 | imputation | 0.742 (0.663-0.831) | 2.33🞨10^-7^ | 0.789 (0.647-0.962) | 1.92🞨10^-2^ | 0.742 (0.661-0.833) | 3.90🞨10^-7^ | 0.819 (0.675-0.993) | 4.22🞨10^-2^ | 1.025 (0.937-1.121) | 5.91🞨10^-1^ | 1.023 (0.874-1.197) | 7.76🞨10^-1^ | 1.016 (0.929-1.112) | 7.26🞨10^-1^ | 1.047 (0.893-1.227) | 5.70🞨10^-1^ |
| rs17482753 | Chip | 0.720 (0.625-0.829) | 5.06🞨10^-6^ | 0.727 (0.561-0.941) | 1.58🞨10^-2^ | 0.727 (0.629-0.839) | 1.44🞨10^-5^ | 0.674 (0.522-0.871) | 2.60🞨10^-3^ | 1.01 (0.905-1.128) | 8.53🞨10^-1^ | 1.062 (0.871-1.294) | 5.51🞨10^-1^ | 0.961 (0.861-1.074) | 4.85🞨10^-1^ | 0.942 (0.768-1.154) | 5.63🞨10^-1^ |
| rs17489282 | imputation | 0.743 (0.664-0.832) | 2.60🞨10^-7^ | 0.789 (0.647-0.961) | 1.87🞨10^-2^ | 0.740 (0.659-0.830) | 3.11🞨10^-7^ | 0.816 (0.673-0.989) | 3.90🞨10^-2^ | 1.027 (0.939-1.123) | 5.63🞨10^-1^ | 1.022 (0.874-1.196) | 7.82🞨10^-1^ | 1.016 (0.929-1.112) | 7.22🞨10^-1^ | 1.046 (0.892-1.226) | 5.77🞨10^-1^ |
| rs1837842 | imputation | 0.741 (0.662-0.829) | 1.98🞨10^-7^ | 0.815 (0.670-0.992) | 4.16🞨10^-2^ | 0.745 (0.664-0.836) | 5.56🞨10^-7^ | 0.821 (0.677-0.994) | 4.41🞨10^-2^ | 1.026 (0.938-1.123) | 5.70🞨10^-1^ | 1.036 (0.886-1.212) | 6.55🞨10^-1^ | 1.018 (0.930-1.113) | 7.03🞨10^-1^ | 1.062 (0.906-1.244) | 4.57🞨10^-1^ |
| rs1919484 | imputation | 0.740 (0.661-0.829) | 1.89🞨10^-7^ | 0.815 (0.670-0.992) | 4.16🞨10^-2^ | 0.745 (0.664-0.836) | 5.45🞨10^-7^ | 0.821 (0.677-0.994) | 4.41🞨10^-2^ | 1.027 (0.939-1.124) | 5.58🞨10^-1^ | 1.036 (0.886-1.212) | 6.55🞨10^-1^ | 1.019 (0.931-1.114) | 6.89🞨10^-1^ | 1.062 (0.906-1.244) | 4.57🞨10^-1^ |
| rs263 | imputation | 0.779 (0.693-0.874) | 2.44🞨10^-5^ | 0.812 (0.665-0.993) | 4.28🞨10^-2^ | 0.778 (0.691-0.875) | 3.19🞨10^-5^ | 0.883 (0.724-1.077) | 2.20🞨10^-1^ | 1.037 (0.945-1.139) | 4.46🞨10^-1^ | 1.046 (0.890-1.229) | 5.84🞨10^-1^ | 0.987 (0.899-1.084) | 7.89🞨10^-1^ | 1.08 (0.917-1.271) | 3.56🞨10^-1^ |
| rs271 | Chip | 0.774 (0.690-0.867) | 1.09🞨10^-5^ | 0.835 (0.687-1.016) | 7.21🞨10^-2^ | 0.778 (0.693-0.875) | 2.60🞨10^-5^ | 0.877 (0.723-1.064) | 1.85🞨10^-1^ | 1.047 (0.955-1.148) | 3.26🞨10^-1^ | 1.059 (0.904-1.241) | 4.75🞨10^-1^ | 0.973 (0.887-1.067) | 5.58🞨10^-1^ | 1.028 (0.876-1.207) | 7.32🞨10^-1^ |
| rs3782889 | Chip | 0.874 (0.779-0.979) | 2.06🞨10^-2^ | 0.865 (0.708-1.058) | 1.58🞨10^-1^ | 1.15 (1.029-1.285) | 1.35🞨10^-2^ | 0.999 (0.824-1.211) | 9.90🞨10^-1^ | 0.862 (0.784-0.948) | 2.22🞨10^-3^ | 0.8671 (0.735-1.022) | 8.91🞨10^-2^ | 0.846 (0.769-0.931) | 6.01🞨10^-4^ | 0.864 (0.731-1.021) | 8.63🞨10^-2^ |
| rs4244457 | Chip |  |  |  |  |  |  |  |  |  |  |  |  |  |  |  |  |
| rs4922117 | imputation | 0.743 (0.664-0.832) | 2.60🞨10^-7^ | 0.789 (0.647-0.961) | 1.87🞨10^-2^ | 0.740 (0.659-0.830) | 3.11🞨10^-7^ | 0.816 (0.673-0.989) | 3.90🞨10^-2^ | 1.027 (0.939-1.123) | 5.63🞨10^-1^ | 1.022 (0.874-1.196) | 7.82🞨10^-1^ | 1.016 (0.929-1.112) | 7.22🞨10^-1^ | 1.046 (0.892-1.226) | 5.77🞨10^-1^ |
| rs6494005 | imputation | 0.972 (0.878-1.077) | 5.94🞨10^-1^ | 0.898 (0.748-1.079) | 2.51🞨10^-1^ | 1.236 (1.118-1.366) | 3.53🞨10^-5^ | 1.068 (0.895-1.275) | 4.64🞨10^-1^ | 0.988 (0.907-1.076) | 7.83🞨10^-1^ | 0.996 (0.857-1.157) | 9.58🞨10^-1^ | 0.955 (0.876-1.04) | 2.88🞨10^-1^ | 1.12 (0.963-1.302) | 1.42🞨10^-1^ |
| rs6586891 | Chip |  |  |  |  |  |  |  |  |  |  |  |  |  |  |  |  |
| **rs6589566** | imputation | **1.482 (1.341-1.637)** | **1.06🞨10^-14^** | **1.522 (1.267-1.828)** | **6.90🞨10^-6^** | 1.178 (1.063-1.305) | 1.76🞨10^-3^ | 1.126 (0.938-1.351) | 2.04🞨10^-1^ | 1.021 (0.936-1.115) | 6.34🞨10^-1^ | 1.107 (0.946-1.295) | 2.04🞨10^-1^ | 0.991 (0.908-1.081) | 8.34🞨10^-1^ | 0.963 (0.821-1.129) | 6.44🞨10^-1^ |
| rs6589567 | imputation | 1.194 (1.073-1.328) | 0.11🞨10^-2^ | 1.444 (1.194-1.747) | 1.54🞨10^-4^ | 1.112 (0.996-1.241) | 5.84🞨10^-2^ | 1.242 (1.031-1.497) | 2.25🞨10^-2^ | 1.032 (0.942-1.13) | 5.02🞨10^-1^ | 1.179 (1.003-1.384) | 4.54🞨10^-2^ | 0.940 (0.858-1.03) | 1.88🞨10^-1^ | 1.01 (0.857-1.191) | 9.01🞨10^-1^ |
| rs7013777 | imputation | 0.745 (0.665-0.834) | 3.57🞨10^-7^ | 0.794 (0.651-0.968) | 2.28🞨10^-2^ | 0.757 (0.675-0.849) | 2.20🞨10^-6^ | 0.794 (0.653-0.965) | 2.06🞨10^-2^ | 1.014 (0.926-1.11) | 7.67🞨10^-1^ | 1.093 (0.933-1.279) | 2.69🞨10^-1^ | 1.016 (0.928-1.112) | 7.31🞨10^-1^ | 1.086 (0.926-1.273) | 3.10🞨10^-1^ |
| rs7396835 | Chip | 1.272 (1.162-1.393) | 1.94🞨10^-7^ | 1.245 (1.061-1.461) | 7.20🞨10^-3^ | 1.152 (1.05-1.263) | 2.65🞨10^-3^ | 1.095 (0.934-1.282) | 2.60🞨10^-1^ | 1.045 (0.967-1.129) | 2.64🞨10^-1^ | 0.913 (0.797-1.045) | 1.85🞨10^-1^ | 1.08 (0.999-1.167) | 5.14🞨10^-2^ | 0.974 (0.850-1.117) | 7.12🞨10^-1^ |
| rs7396851 | imputation | 1.269 (1.159-1.39) | 2.52🞨10^-7^ | 1.24 (1.057-1.455) | 8.34🞨10^-3^ | 1.158 (1.056-1.27) | 1.79🞨10^-3^ | 1.102 (0.941-1.291) | 2.28🞨10^-1^ | 1.052 (0.973-1.136) | 2.03🞨10^-1^ | 0.918 (0.802-1.05) | 2.13🞨10^-1^ | 1.076 (0.996-1.163) | 6.22🞨10^-2^ | 0.972 (0.848-1.115) | 6.93🞨10^-1^ |
| rs7461115 | imputation | 0.749 (0.668-0.839) | 6.08🞨10^-7^ | 0.796 (0.653-0.971) | 2.46🞨10^-2^ | 0.745 (0.664-0.837) | 7.62🞨10^-7^ | 0.800 (0.658-0.973) | 2.55🞨10^-2^ | 1.019 (0.930-1.116) | 6.88🞨10^-1^ | 1.089 (0.931-1.275) | 2.87🞨10^-1^ | 1.023 (0.934-1.12) | 6.28🞨10^-1^ | 1.102 (0.939-1.292) | 2.33🞨10^-1^ |
| rs765547 | imputation | 0.740 (0.661-0.829) | 1.81🞨10^-7^ | 0.815 (0.670-0.992) | 4.16🞨10^-2^ | 0.745 (0.664-0.835) | 5.34🞨10^-7^ | 0.821 (0.677-0.994) | 4.41🞨10^-2^ | 1.027 (0.939-1.123) | 5.64🞨10^-1^ | 1.036 (0.886-1.212) | 6.55🞨10^-1^ | 1.019 (0.931-1.115) | 6.76🞨10^-1^ | 1.062 (0.906-1.244) | 4.57🞨10^-1^ |
| rs16940170 | imputation | 1.033 (0.938-1.137) | 5.09🞨10^-1^ | 0.914 (0.770-1.085) | 3.05🞨10^-1^ | 0.793 (0.717-0.878) | 7.81🞨10^-6^ | 0.705 (0.592-0.840) | 9.51🞨10^-5^ | 1.006 (0.928-1.091) | 8.82🞨10^-1^ | 1.003 (0.872-1.154) | 9.64🞨10^-1^ | 0.931 (0.858-1.011) | 8.90🞨10^-2^ | 0.929 (0.805-1.071) | 3.12🞨10^-1^ |
| rs16940212 | Chip | 1.037 (0.945-1.137) | 4.41🞨10^-1^ | 0.936 (0.795-1.102) | 4.27🞨10^-1^ | 0.796 (0.723-0.877) | 4.15🞨10^-6^ | 0.731 (0.619-0.864) | 2.48🞨10^-4^ | 1.016 (0.94-1.098) | 6.87🞨10^-1^ | 0.998 (0.872-1.142) | 9.76🞨10^-1^ | 0.943 (0.872-1.02) | 1.44🞨10^-1^ | 0.898 (0.782-1.03) | 1.25🞨10^-1^ |
| rs17482310 | imputation | 0.748 (0.647-0.864) | 8.16🞨10^-5^ | 0.727 (0.559-0.945) | 1.72🞨10^-2^ | 0.742 (0.640-0.861) | 8.144🞨10^-5^ | 0.667 (0.512-0.868) | 2.59🞨10^-3^ | 0.989 (0.883-1.109) | 8.59🞨10^-1^ | 1.044 (0.854-1.278) | 6.72🞨10^-1^ | 0.934 (0.833-1.048) | 2.48🞨10^-1^ | 0.974 (0.793-1.196) | 8.02🞨10^-1^ |
| rs486394 | imputation | 1.278 (1.109-1.472) | 7.02🞨10^-4^ | 1.452 (1.135-1.858) | 3.04🞨10^-3^ | 1.103 (0.952-1.278) | 1.90🞨10^-1^ | 1.107 (0.857-1.429) | 4.35🞨10^-1^ | 0.998 (0.881-1.132) | 9.83🞨10^-1^ | 1.061 (0.854-1.318) | 5.95🞨10^-1^ | 0.994 (0.877-1.127) | 9.29🞨10^-1^ | 0.926 (0.741-1.157) | 5.01🞨10^-1^ |
| rs495348 | imputation | 1.036 (0.941-1.14) | 4.72🞨10^-1^ | 0.929 (0.784-1.102) | 3.99🞨10^-1^ | 0.794 (0.718-0.878) | 7.60🞨10^-6^ | 0.717 (0.603-0.853) | 1.77🞨10^-4^ | 1.011 (0.932-1.096) | 7.91🞨10^-1^ | 0.997 (0.867-1.146) | 9.63🞨10^-1^ | 0.928 (0.856-1.007) | 7.44🞨10^-2^ | 0.916 (0.794-1.056) | 2.26🞨10^-1^ |
| **rs2303790** | Chip | 1.144 (0.932-1.405) | 1.97🞨10^-1^ | 0.984 (0.690-1.403) | 9.30🞨10^-1^ | **0.349 (0.255-0.478)** | **5.31🞨10^-11^** | **0.409 (0.256-0.653)** | **1.81🞨10^-4^** | 1.062 (0.890-1.267) | 5.03🞨10^-1^ | 0.91 (0.679-1.22) | 5.29🞨10^-1^ | 0.996 (0.834-1.191) | 9.70🞨10^-1^ | 1.222 (0.912-1.635) | 1.79🞨10^-1^ |
| rs5880 | Chip |  |  |  |  |  |  |  |  |  |  |  |  |  |  |  |  |
| rs5881 | Chip |  |  |  |  |  |  |  |  |  |  |  |  |  |  |  |  |

rs number, SNP ID in dbSNP database; HDL, high density lipoprotein; FBG, fasting blood glucose; BP, blood pressure; OR, odds ratio; CI, confidence interval, respectively
