# Supplementary figures and images for: Genome-wide association study of metabolic syndrome in Korean populations
Source: PLoS One. 2020 Jan 7;15(1):e0227357. doi: 10.1371/journal.pone.0227357 (PMC6946588; doi:10.1371/journal.pone.0227357)

**S1 Fig. Principal component analysis (PCA) for our Korean population and 1000 genome phase 3 data**

**
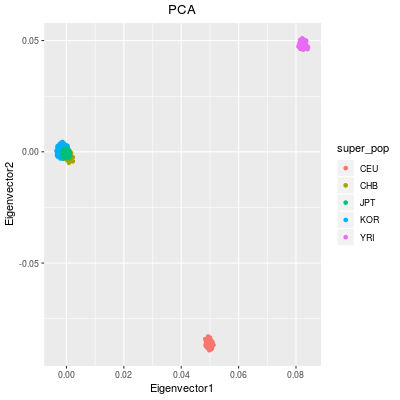
**

Supplement: S1 Fig — (DOCX) [file pone.0227357.s003.docx]
