# Supplementary material for: Genome-wide association study of metabolic syndrome in Korean populations
Source: PLoS One. 2020 Jan 7;15(1):e0227357. doi: 10.1371/journal.pone.0227357 (PMC6946588; doi:10.1371/journal.pone.0227357)

**S3 Fig. Quantile-quantile Plots of the Association Test Results for Metabolic Syndrome Components**

(A) Hypertriglyceridemia (Discovery set)


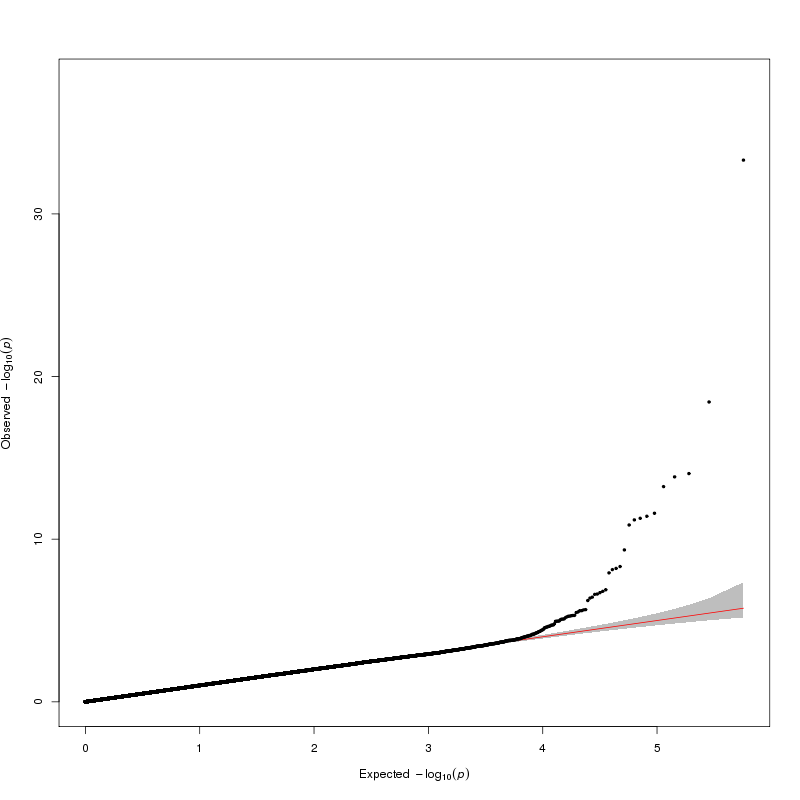


(B) Hypertriglyceridemia (Replication set)


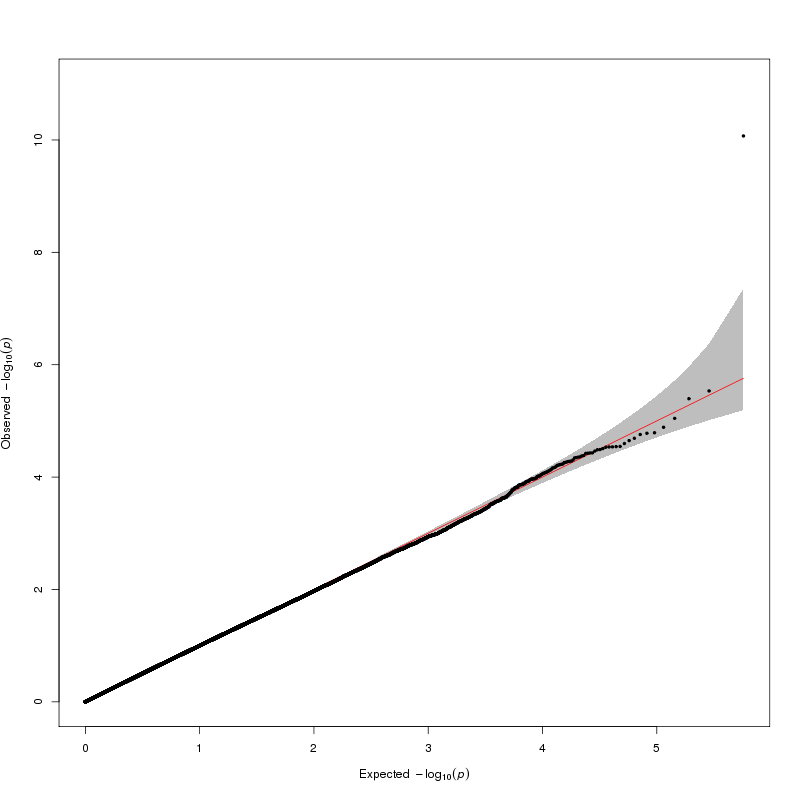


(C) Low HDL-C (Discovery set)


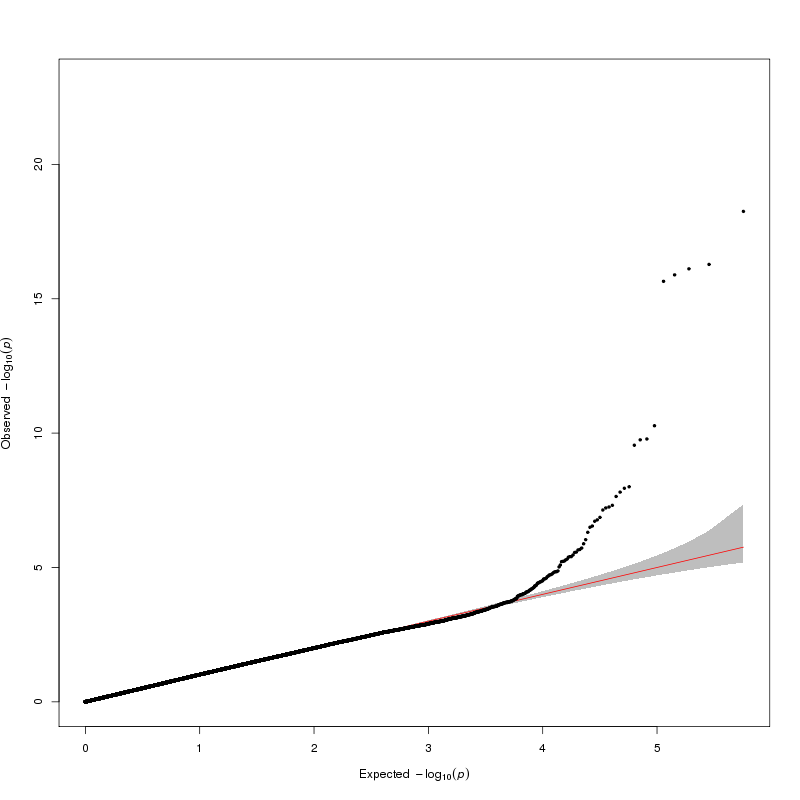


(D) Low HDL-C (Replication set)


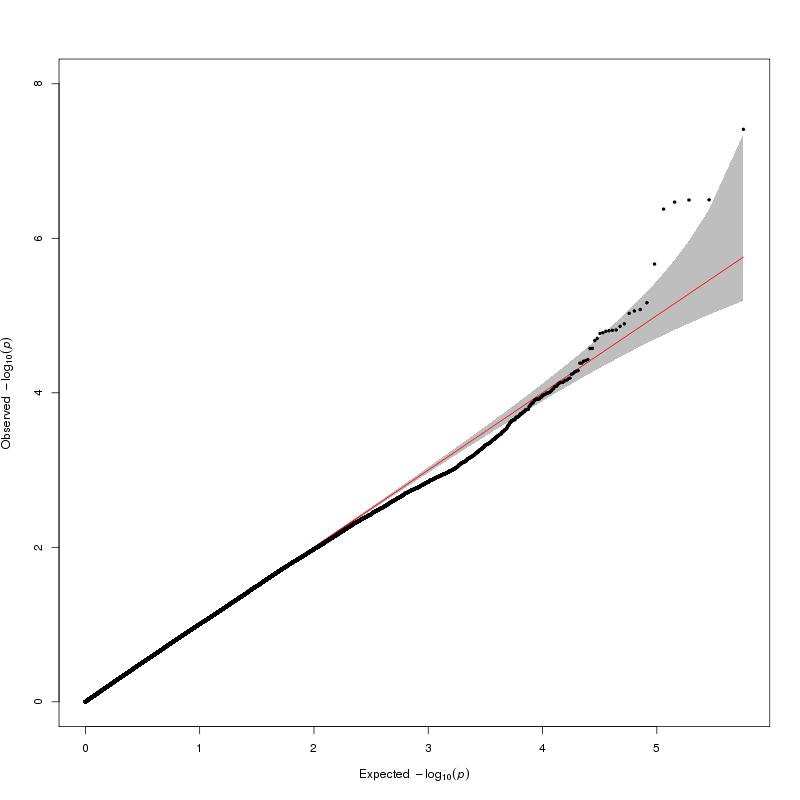


(E) High Fasting Blood Glucose (Discovery set)


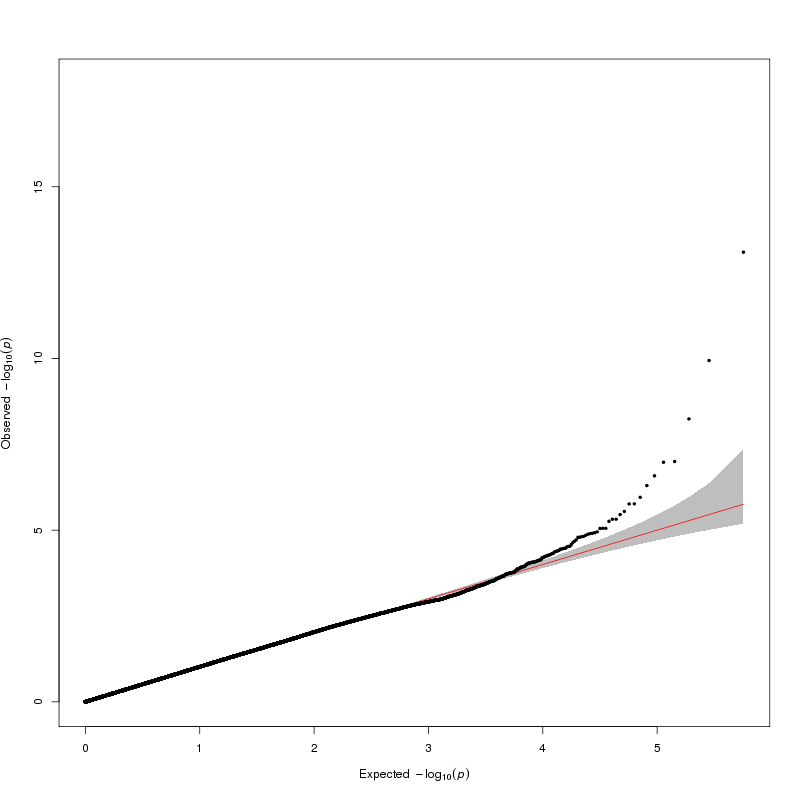


(F) High Fasting Blood Glucose (Replication set)


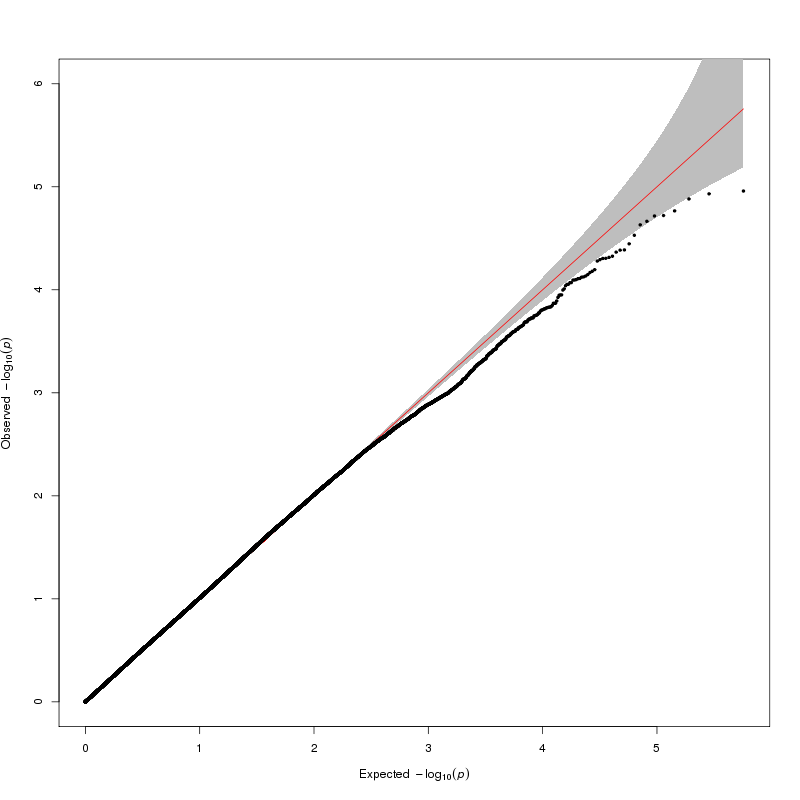


(G) High Blood Pressure (Discovery set)


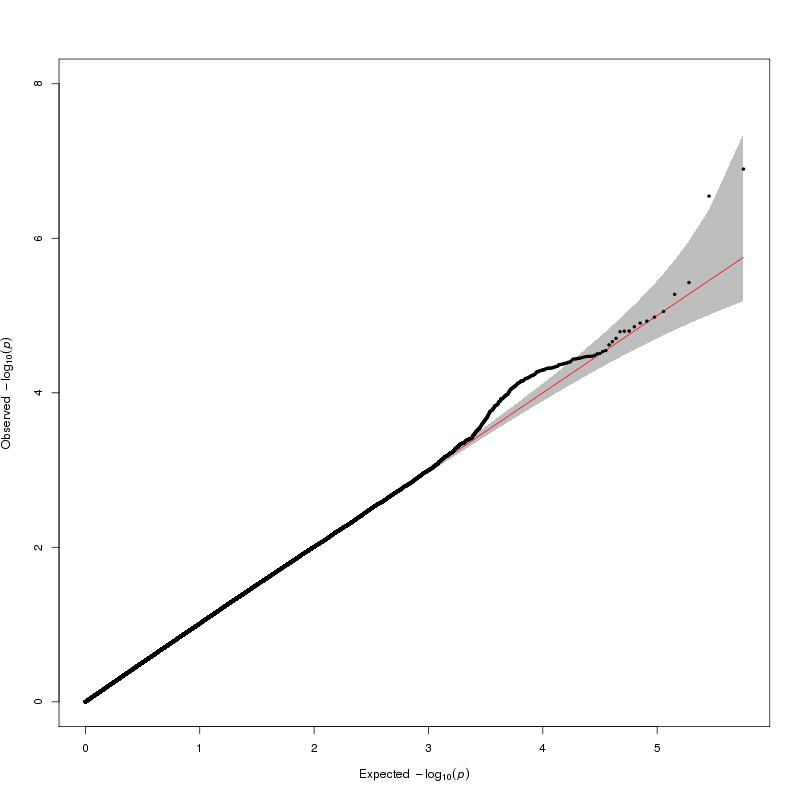


(H) High Blood Pressure (Replication set)


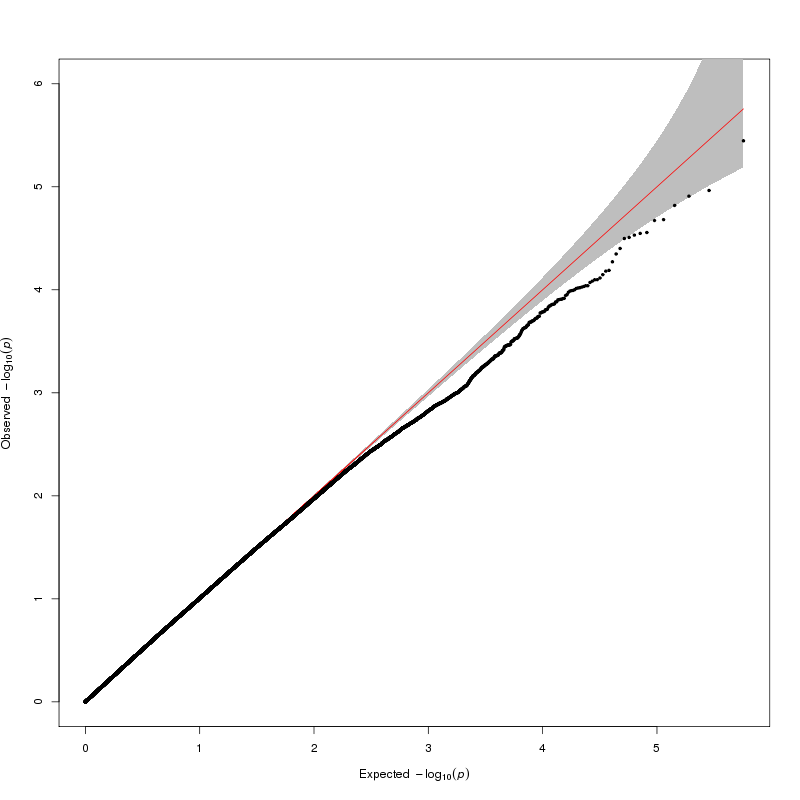

Supplement: S3 Fig — (DOCX) [file pone.0227357.s005.docx]
