# Supplementary material for: Genome-wide association study of metabolic syndrome in Korean populations
Source: PLoS One. 2020 Jan 7;15(1):e0227357. doi: 10.1371/journal.pone.0227357 (PMC6946588; doi:10.1371/journal.pone.0227357)
Supplement: S1 Appendix — (DOCX) [file pone.0227357.s006.docx]

**S1 Appendix. The questionnaire for study subjects.**

(A) English translated version

1. What is the reason for this medical examination?

○ Routine checkup

○ Checkup for the work or group

○ Recent health problems.

▶ Mark ● accordingly

○ Weight loss (For recent 2~3 months, losing 10% of the body weight)

○ Digestive symptoms (Indigestion, abdominal pain, NauseaㆍVomiting, Heartburn, Black & Bloody stools, Bowel habit change)

○ Respiratory & Allergy symptoms (SneezeㆍCoughㆍRunny nose, Wheezing, Coughing over 3 weeks)

○ Cardiovascular symptoms (Dyspnea or Chest pain when exercising or with activities, Palpitation)

○ Nervous symptoms (Headache, Syncope, Dizziness, Memory loss)

▶ Write other symptoms below

1)

2)

3)

2. Have you ever done colonoscopy?

○ No

○ Yes ▶ The most recent year: Year

▶ Have you removed colonic polyp before? ○ No ○ Yes

3. Have you ever had any side effects from contrast media during CT tests? ○ No ○ Yes

4. Do you have any allergies? ○ No ○ Yes

▶ If yes, what is the cause of the allergy?

○ Drugs (Antibiotics, Local anesthetics, Anti-inflammatory)

○ Allergens (House Dust, Pollen, Food)

5. If you have been diagnosed with any of the diseases below, mark ● accordingly.

**○ Not applicable to the below diagnosis**

| Diagnosis | Diagnosed | Medication | Remark |
| --- | --- | --- | --- |
| Hypertension | ○ | ○ |  |
| Diabetes | ○ | ○ |  |
| Hyperlipidemia | ○ | ○ |  |
| AnginaㆍMI | ○ | ○ | ○Angioplasty/Stent ○Operation |
| CVA (Stroke) | ○ | ○ |  |
| Chronic Renal disease | ○ | ○ | ○Decreased renal function ○dialysis |
| Gastric/Duodenal ulcer | ○ | ○ |  |
| Chronic Hepatitis B | ○ | ○ |  |
| Chronic Hepatitis C | ○ | ○ |  |
| Lung Tuberculosis | ○ | ○ | ○Treatment completed ○Scar left |
| Bronchial Asthma | ○ | ○ |  |
| Allergic Rhinitis | ○ | ○ |  |
| Fractures | ○ | Sites: | |
| Others Diseases | ○ | Diagnosis: | |
| Cancer | ○Lung ○Stomach ○ColonㆍSigmoid ○Liver ○Breast  ○Cervix ○Thyroid ○Prostate  ○Other: _______________________________________________________ | | |
| Abdominal Surgery | Lesion: ○StomachㆍDuodenum ○ColonㆍSigmoid ○Appendix  ○Gall bladder ○Uterus (except D&C) ○OvaryㆍFallopian tube  Diagnosis:  __________________________________________________________ | | |
| Other surgery | Lesion:  __________________________________________________________  Diagnosis:  __________________________________________________________ | | |

6. If you are currently taking any of the medication below more than twice a week, mark ● accordingly.

**○ Not applicable to the below medication**

| Medication | Medication |
| --- | --- |
| ○ Aspirin | ○ Osteoporosis (once a week/ month/ year) |
| ○ Anti-coagulant (WarfarinㆍCumadine) | ○ Calcium |
| ○ Anti-inflammatory | ○ SedativesㆍSleeping Pills |
| ○ Steroids | ○ Oriental Herbal Medication |
| ○ Thyroid Hormone Agent (Thyroxine) | ○ Others ( ) |
| ○ Antithyroid Agent (Methimazole) |  |

7. Have ever treated Helicobacter pylori infection?

○ No ○ Yes ▶ ○ Bacteriostat treatment succeeded ○ Bacteriostat treatment failed ○ Do not know

8. Have any of your family members (parents, sibling or children) been diagnosed or died with any of the diseases below?

| Diagnosis | Yes | Diagnosis | Yes |
| --- | --- | --- | --- |
| Hypertension | ○ | Chronic hepatitisㆍLiver cirrhosis | ○ |
| Diabetes | ○ | Liver Cancer | ○ |
| AnginaㆍMI (If diagnosed under 45 years (male), 55 years (female) old) | ○ | Stomach Cancer | ○ |
|  |  | ColonㆍSigmoid Cancer | ○ |
| CVA (Stroke) | ○ | Lung Cancer | ○ |

9. Have you ever smoked more than 5 packs (100 cigarettes)?

○ No (☞ Go question no. 10)

○ If smoked before, but quit

| How long has it been since you quit? | year(s) |
| --- | --- |
| How long have you been smoking before quitting? | year(s) |
| How much did you smoke a day before quitting? | Cigarette(s) |

○ If currently smoking

| How long have you been smoking? | year(s) |
| --- | --- |
| How much do you smoke a day? | cigarette(s) |

10. How often do you drink alcohol?

Per month ○ less than once ○ 2~4 times

Per week ○ twice ○ 3 times ○ 4 times ○ 5 times ○ 6 times ○ 7 times

▶ How much do you usually drink at a time?

(※ Count the each glass of the type of alcohol. ex, beer 1 can (355cc) = beer 1.6 glasses)

○1-2 glass(es) ○ 3-4 glasses ○ 5-6 glasses ○ 7-9 glasses ○ more than 10 glasses

11-1. **During the last one week**, how many days did you have **the intense activities** over 10 min. per day that make you very breathless? (Ex: running, Aerobic dance, Fast cycling, Squash, Climbing, Carrying heavy things, etc)

○ 0 ○ 1 ○2 ○ 3 ○ 4 ○ 5 ○ 6 ○ 7

▶ How long do you have the intense activities averagely? Minute(s)/day

11-2. **During the last one week**, how many days did you have **the moderate activities** over 30 min. per day that make you breathless? (Ex: Normal cycling, Tennis, Carrying light things, Kneeling down and mopping the floor, etc/ Walking is not included)

○ 0 ○ 1 ○2 ○ 3 ○ 4 ○ 5 ○ 6 ○ 7

▶ How long do you have the moderate activities averagely? Minute(s)/day

11-3. **During the last one week**, how many days did you **walk more than 10 min**. at a time? (It is included not only the walk to use public transport but also the walk during leisure activities)

○ 0 ○ 1 ○2 ○ 3 ○ 4 ○ 5 ○ 6 ○ 7

▶ How long do you walk averagely? Minute(s)/day

(B) Original Korean version
